# Feeling Blue and Getting Red: An Exploratory Study on the Effect of Color in the Processing of Emotion Information

**DOI:** 10.3389/fpsyg.2022.515215

**Published:** 2022-06-30

**Authors:** June Kang, Yeo Eun Park, Ho-Kyoung Yoon

**Affiliations:** ^1^Department of Brain and Cognitive Engineering, Korea University, Seoul, South Korea; ^2^Department of Psychiatry, College of Medicine, Korea University, Seoul, South Korea

**Keywords:** emotion, color, Stroop, emotional word, facial expression, priming

## Abstract

Specific emotions and colors are associated. The current study tested whether the interference of colors with affective processing occurs solely in the semantic stage or extends to a more complex stage like the lexical processing of emotional words. We performed two experiments to determine the effect of colors on affective processing. In Experiment 1, participants completed a color-emotion priming task. The priming stimulus included a color-tinted (blue, red, and gray) image of a neutral face, followed by a target stimulus of gray-scaled emotional (angry and sad) and neutral faces after 50 ms. Experiment 2 used a modified emostroop paradigm and superimposed emotion words on the center of the color-tinted emotional and neutral faces. Results showed the priming effect of red for the angry face compared to the control, but not in blue for the sad face compared to the control. However, responses to the blue-sad pair were significantly faster than the red-sad pair. In the color-emostroop task, we observed a significant interaction between color and emotion target words in the modified emostroop task. Participants detected sad targets more accurately and faster in blue than red, but only in the incongruent condition. The results indicate that the influence of color in the processing of emotional information exists at the semantic level but found no evidence supporting the lexical level effect.

## Introduction

The emotional meaning of colors varies across cultures (Hupka et al., 1997). Nevertheless, some colors seem to have certain degrees of universal emotional nuances across cultures (Odbert et al., 1942; Adams and Osgood, 1973; Hupka et al., 1997). Several studies have reported these commonalities. For example, Germany, Mexico, Poland, Russia, and the United States associate red with anger (Byrnes, 1983; Hupka et al., 1997; Clarke and Costall, 2008; Fetterman et al., 2010). Similarly, many countries associate blue with sadness (Byrnes, 1983; Levy, 1984; Clarke and Costall, 2008; Hanada, 2018; Takahashi and Kawabata, 2018). We also find similar associations between emotions and colors in Korean culture. For example, Chǒng Chisang’s (~1,135) classic poem “Song-In (Seeing off),” associates blue with the sorrow of farewell: “別淚年年添綠波 (Tears at parting each year add more blue swells).” Korean literature also commonly finds an association between red and anger. For instance, the Korean poet Park JongHwa (1901 ~ 1981) wrote, “격념(激念)에 뛰는 빨간 염통이 터져 아름다운 피를 뽐고 넘어질 때까지 힘껏 성내어 보아라 (Boast the rage till the red heart bursts the beautiful blood and falls).”

If colors connote emotion meaning, the color should influence emotion information processing. Thus, a few studies empirically tested the association between color and emotion (Moller et al., 2009; Kuhbandner and Pekrun, 2013). For example, Moller et al. (2009) reported the priming effect of colors in a word categorization task using negative (failure-related) and positive (success-related) words. Participants’ reduced their response times when the researchers presented negative words in red and positive words in green. The researchers observed this effect for adjectives and nouns. Another study investigating the effect of color on participants’ memories of emotional words reported corresponding results. Participants recalled negative words better when presented in red, whereas positive words received a better recall in green (Kuhbandner and Pekrun, 2013).

These findings support the effect of colors in the processing of emotion stimuli. However, whether the effect relates to semantic or more elaborated levels like lexical processing is unclear because people process emotions at semantic and conceptual (lexical) levels (Palermo and Rhodes, 2007; Scherer and Moors, 2019). Similarly, both automatic processing levels can engage color information when presented below the threshold of conscious detection (Ro et al., 2009), and a more conceptual level in word decision tasks (MacLeod, 1991; Heurley et al., 2013) even when trying to attend the physical-level information of the colored word (MacLeod, 1991). Thus, colors may interfere at various processing levels.

The current study aimed to test whether the interference of colors with affective processing occurs solely in the semantic stage or in more conceptual stage like the lexical processing of emotional word. To examine the above idea, we adopted the color-emotion priming task in Experiment 1 and modified version of the emostroop task (Preston and Stansfield, 2008) in Experiment 2. Priming refers the phenomenon that the presentation of the preceding priming stimulus alters the perception of the target stimulus (Murphy and Zajonc, 1993). In Experiment 1, the nonverbal stimuli-facial expressions of emotions-presented as target. In contrast, the participants respond to the emotion word superimposed on color-tinted facial expression, which requires lexical level processing. Therefore, the interference in Experiment 2 may indicate the lexical level effect of colors in emotion processing.

The original Stroop task was developed to study interference between automatic and controlled process (Stroop, 1935; Washburn, 2016). In the original Stroop task, the participant asked to name the color of the presented words. Since reading is an automatic process, it is hard to suppress. Therefore, the response time slows in the incongruent condition which requires naming the color mismatch to the meaning of the word. Numerous “Emotional Stroop” tasks have been developed, based on the idea that the emotional processing is also difficult to suppress. The emostroop task (Preston and Stansfield, 2008) is one of the variants. In the emostroop task, the emotion words superimposed on emotional faces, and the participants asked to categorize the emotion words. Like the Stroop task, it is expected that the interference between the emotion information of faces and words leads to a slower response time in the incongruent condition compare to the congruent condition. Although the background face is irrelevant to the task, the interference occurs because the face rapidly processes at a conceptual level. The current study added color information *via* blue and red tints on the stimuli of the emostroop task.

We hypothesized that if the lexical level interference of the color on emotion exists, the reaction time to emotional word will be faster when it is presented with the associated color, compare to not associated color. In the similar vein, the accuracy would be higher when the target word is presented with the associated color (Table 1).

**Table 1:** The contingency table of color-emostroop task.

Condition	Target	Hypothesis
Incongruent	Angry	If lexical level effect of Red on Angry exists, Accuracy: Red > Blue, RT: Red < Blue
	Happy	None
	Sad	If lexical level effect of Blue on Sad exists, Accuracy: Blue > Red, RT: Blue < Red

Thus, Experiment 1 measured the priming effect of colors of preceding neutral faces on the response time toward target emotional faces. Experiment 2 assessed the impact of color on the Emostroop effect (slower reaction time to classify incongruent adjectives compared to congruent ones) between associated and non-associated colors.

We counterbalanced the order of experiments over participants to avoid the order effect. In addition, there was a five-minute rest period between experiments.

## Experiment 1

### Method

#### Participants

Using an online application system, we recruited 40 healthy, right-handed participants (16 male, 24 female) from local universities (mean age = 25.50; SD = 3.57). All participants reported no history of psychiatric illness and had normal or corrected to normal vision. We used the Ishihara test (Ishihara, 1917) to evaluate color vision. After a complete explanation of the study to all participants, we obtained their written informed consent. The Institutional Review Board of Korea University approved this study, approval number KUIRB14215A2. We conducted this study according to the Declaration of Helsinki as revised in 1989.

#### Apparatus

We tested participants individually in a dark room. We presented the stimuli on a 17-inch FlexScan L768 monitor (250 cd/m2, 1,000:1, EIZO Corporation, Ishikawa, Japan) in 60 Hz with PsychoPy software (Peirce, 2007). We recorded forced-choice responses using an RB-730 response pad (Cedrus, San Pedro, CA), labeled as “sad,” “neutral,” and “angry” on the third, fourth, and fifth buttons. In addition, we counterbalanced the button order between participants. We used the PR-650 spectroradiometer (Photo Research, Inc., Chatsworth, CA) and PsychoPy’s built-in calibration module to color calibrate and gamma correct the monitor.

### Stimuli and Procedure

#### Colored Facial Expressions

We selected emotional and neutral facial expression stimuli from the Korean Facial Expressions of Emotion (KOFEE) database (Park et al., 2011). The KOFEE comprises still images of seven facial expressions (happy, disgusted, angry, sad, surprised, fearful, and contemptuous) and neutral faces of Korean models. Researchers trained the models to contract and relax different facial muscles (action units) associated with each emotion based on the Facial Action Coding System (FACS; Ekman et al., 1983). We validated every facial expression in the database through subsequent FACS coding. In Experiment 1, we took emotional (angry and sad) and neutral stimuli from model SR in the database. We matched the luminance of each stimulus to 100 cd/m^2^ using the PR-650 spectrophotometer (Photo Research, Burbank, CA).

#### Color Priming

**Figure 1:**
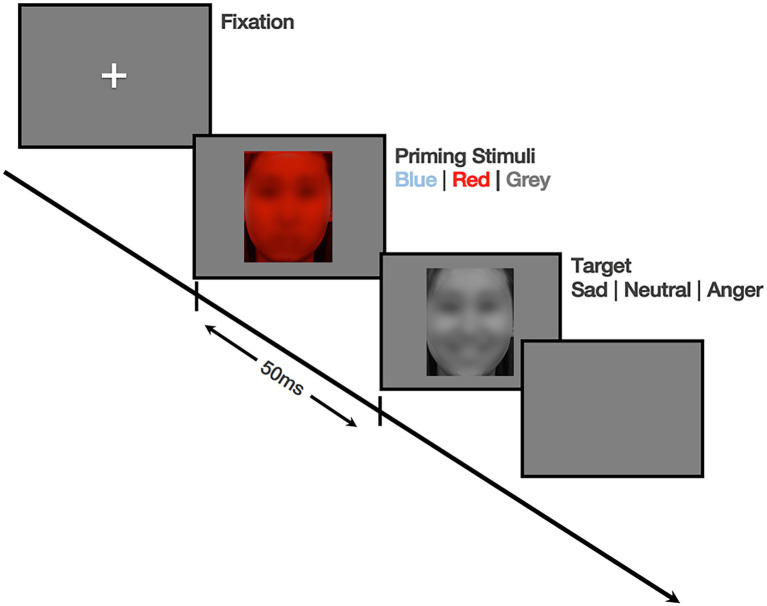
Sequence of events for Experiment 1 (The face in the figure is selected from example photos of KOFEE and reprinted under permission based on consent of initial development study). The face is blurred following the ICMJE recommendations.

In each trial, we presented a central fixation cross for 500 ms. Then we introduced the color-tinted image (blue, red, and gray) of the model’s neutral face as the priming stimulus for 50 ms followed by gray-scaled emotional (angry and sad) and neutral faces for 50 ms as target images. The task consisted of 90 trials with a one-second inter-trial interval (ITI) and took about 5 min. We counterbalanced all trials to prevent an order effect, and we tested participants individually in a dark room. We instructed them to fixate on the targets and respond by pressing the key that matched the facial expressions as fast and accurately as possible. The participants responded *via* key presses that matched the emotions of the target faces. Figure 1 provides details of this experiment.

### Statistical Analysis

First, we examined the effects using a repeated-measures analysis of variance (ANOVAs) with “color of primer” and “emotion of target” as within-subject factors. We considered *p* < 0.05 statistically significant. We conducted all statistical analyses using IBM SPSS Statistics for Macintosh (SPSS 23; IBM Corp, Armonk, NY).

### Results

Table 2 provides the results of the color priming task (Experiment 1). The participants responded to the emotion of the target faces.

**Table 2:** Statistical results of repeated measures ANOVAs in experiment 1 (color priming task).

Effects	Num df	Den DF	*F*	*p*	Partial *η*^2^
**Response Time**					
Priming Color	2	78	4.329	0.016^*^	0.100
Target Emotion	2	78	51.469	<0.001^***^	0.569
Priming Color × Target Emotion	4	156	16.832	<0.001^***^	0.301
**Accuracy**					
Priming Color	2	78	8.518	<0.001^***^	0.179
Target Emotion	2	78	13.562	<0.001^***^	0.258
Priming Color × Target Emotion	4	156	5.905	<0.001^***^	0.131

* *p* < 0.05,

^**^p < 0.01, and

*** *p* < 0.001.

#### Response Time

A 3 (color of prime: gray, red, or blue) × 3 (valence of target face: neutral, angry, or sad) within-subjects ANOVA revealed a statistically significant main effect of priming stimuli [*F*(2,78) = 4.329, *p* = 0.016, partial *η*^2^ = 0.100] and target emotions [F(2,78) = 51.469, *p* < 0.001, partial *η*^2^ = 0.569] on response time. The interaction between the colors of the prime and target emotions was also statistically significant [*F*(4,156) = 16.832, *p* < 0.001, partial *η*^2^ = 0.301].

**Figure 2:**
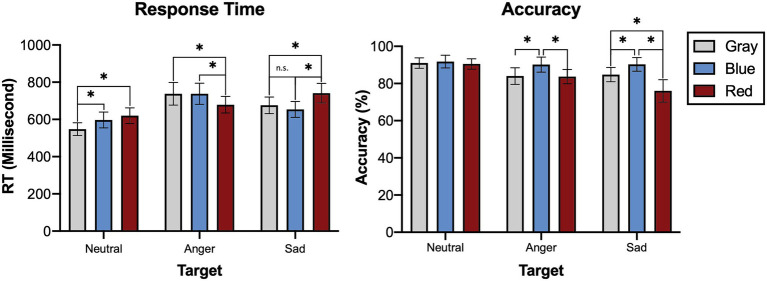
Accuracy and response time for Experiment 1. Bars indicate 95% CIs of means. ^*^p < 0.05; ^**^p < 0.01; and ^***^p < 0.001.

The post-hoc pairwise comparison (Bonferroni-corrected *t*-tests) revealed that the response to the angry face primed by red was significantly faster than the control (gray) condition (*t* = 3.278, *p* = 0.002), and blue condition (*t* = 2.682, *p* = 0.012; Figure 2). The priming effect of blue compared to gray toward the sad face was not statistically significant (*t* = 1.917, *p* = 0.057), but the response was significantly faster when primed by blue than red (*t* = 6.214, *p* < 0.001). We observed a slower response time toward a neutral face when it was preceded by red (*t* = 5.462, *p* < 0.001) and blue (*t* = 4.800, *p* < 0.001), but we observed no difference between the red and blue conditions (*t* = 1.643, *p* = 0.112).

#### Accuracy

A 3 (color of prime: gray, red or blue) × 3 (valence of target face: neutral, angry, or sad) within-subjects analysis of variance (ANOVA) revealed a statistically significant main effect of the priming stimuli [*F*(2,78) = 8.518, *p* < 0.001, partial *η*^2^ = 0.179] and target emotions [F(2,78) = 13.562, *p* < 0.001, partial *η*^2^ = 0.258] on accuracy. The interaction between the colors of prime and target emotions was also statistically significant [*F*(4,156) = 5.905, *p* < 0.001, partial *η*^2^ = 0.131].

In the post-hoc pairwise comparison (Bonferroni-corrected t-tests), the response to an angry face was not significantly more accurate in the red than gray condition (*t* = 0.120, *p* = 0.921, Figure 2). In contrast, the response to a sad face was significantly more accurate in the blue than gray condition (*t* = 2.500, *p* = 0.017). However, the responses to an angry (*t* = 2.241, *p* = 0.029) and sad face (*t* = 4.057, *p* < 0.001) were more accurate when preceded by blue than red.

#### Speed–Accuracy Trade-Off

A correlation between overall speed and accuracy (speed–accuracy trade-off) was not significant (*r* = −0.028, *p* = 0.863).

## Experiment 2

### Method

#### Participants

The same participants from Experiment 1 participated in Experiment 2 after a five-minute rest period.

#### Apparatus

We tested the participants in the same environment as Experiment 1, except we changed the labels on the response pad to “sad,” “happy,” and “angry” on the third, fourth, and fifth buttons. We counterbalanced the button order between the participants.

### Stimuli and Procedure

#### Colored Facial Expressions

We selected the images of facial expressions from KOFEE (Park et al., 2011). In Experiment 2, we used three emotional (happy, angry, and sad) and neutral stimuli of four models (DB, DY, EW, SR; two males and two females). The faces had a red or blue tint for each stimulus. We matched the luminance of each stimulus to 100 cd/m^2^ using the PR-650 spectrophotometer (Photo Research, Burbank, CA).

#### Emotional Word Stimuli

We selected the emotion words for the color-emostroop task from the list of Korean Emotion Terms (Park and Min, 2005). Following the initial emostroop study (Preston and Stansfield, 2008), we selected eight emotion words per three emotions (happy, angry, and sad), matching the valence and arousal ratings to English word stimuli (Shaver et al., 1987).

#### Emostroop Task

**Figure 3:**
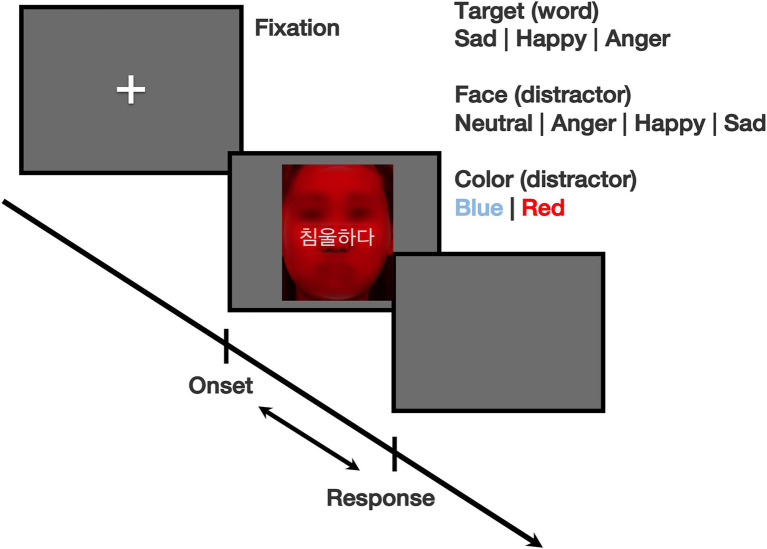
Sequence of events for Experiment 2 (The face in the figure is selected from example photos of KOFEE and reprinted under permission based on consent of initial development study). The face is blurred following the ICMJE recommendations.

In the color-emostroop task, we instructed participants to inhibit their tendency to name the facial expression and instead select an adjective for the emotion superimposed on the center of the facial expression stimuli with 75% opacity. We created 768 combinations (color × facial expression × emotional word) of stimuli pictures. Each stimulus was either congruent or incongruent. In the congruent condition, we matched the target words to the faces. In the incongruent condition, target words were different from the emotion of the faces. We randomized the stimulus order. According to the original emostroop task (Preston and Stansfield, 2008), researchers presented the stimulus until the participant responded. The researchers measured the participants’ reaction time from the onset of the stimulus to the response time. Figure 3 depicts the details. The emostroop task consisted of 768 trials with 500 ms ITI and took about 30 min to complete.

In addition, we tested participants individually in a dark room. We instructed them to fixate on the target and respond by pressing the key that matched the emotional target word as fast and accurately as possible.

### Statistical Analysis

First, we tested the general emostroop effect (slower reaction time to classify emotion adjectives in incongruent than congruent time) and the emostroop effect by color using paired-sample *t*-tests. Then we examined the effects of each factor on speed and accuracy using repeated-measures ANOVAs with the color of image, congruency, and emotion of target word as within-subject factors. We considered *p* < 0.05 statistically significant.

### Results

**Figure 4:**
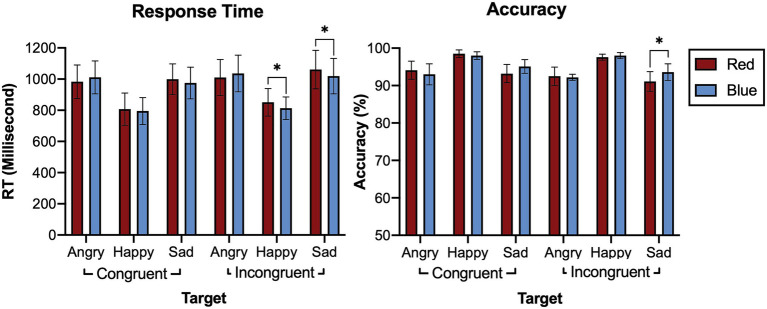
Accuracy and response time for Experiment 2. Bars indicate 95% CIs of means. ^*^p < 0.05; ^**^p < 0.01; and ^***^p < 0.001.

Table 3 and Figure 4 provide the results of the emostroop task (Experiment 2). The participants responded to the emotion meaning of the target words. We observed a significant general emostroop effect in response time with a slower response in the incongruent condition than the congruent condition (*t* = 5.753, *p* < 0.001). The effects in response time were significant in the both blue (*t* = 4.220, *p* < 0.001) and red (*t* = 4.941, *p* < 0.001) conditions. We also observed the effects in accuracy in general (*t* = 5.251, *p* < 0.001), including the blue (*t* = 3.033, *p* = 0.004) and red conditions (*t* = 6.634, *p* < 0.001).

**Table 3:** Statistical results of repeated measures ANOVAs in experiment 2 (color emostroop task).

Effects	Num df	Den DF	*F*	*p*	Partial *η*^2^
**Response Time**					
Color	1	39	1.566	0.218	0.039
Congruency	1	39	11.168	0.002^**^	0.223
Emotion of Target Word	2	78	35.757	<0.001^***^	0.478
Color × Congruency	1	39	0.923	0.343	0.023
Color × Emotion of Target Word	2	78	4.687	0.012^*^	0.106
Congruency × Emotion of Target	2	78	0.652	0.524	0.016
Color × Congruency × Emotion of Target Word	2	78	0.122	0.885	0.003
**Accuracy**					
Color	1	39	2.669	0.110	0.064
Congruency	1	39	11.988	0.001^**^	0.235
Emotion of Target Word	2	78	12.448	<0.001^***^	0.242
Color × Congruency	1	39	2.908	0.096	0.069
Color × Emotion of Target Word	2	78	8.590	<0.001^***^	0.180
Congruency × Emotion of Target	2	78	2.171	0.121	0.053
Color × Congruency × Emotion of Target Word	2	78	0.062	0.940	0.002

* *p* < 0.05,

** *p* < 0.01, and

*** *p* < 0.001.

#### Response Time

A 2 (color: red or blue) × 2 (congruency: congruent or incongruent, happy, or sad) × 3 (valence of target word: angry, happy, or sad) within-subjects ANOVA revealed no statistically significant main effects of color [*F*(1,39) = 1.566, *p* = 0.218, *η*^2^ = 0.039] for response time. In contrast, the main effects of congruency [*F*(1,39) = 11.168, *p* = 0.002, *η*^2^ = 0.223] and target word emotion were statistically significant [*F*(2,78) = 35.757, *p* < 0.001, *η*^2^ = 0.478]. The interactions effect between color and congruency [*F*(1,39) = 0.923, *p* = 0.343, *η*^2^ = 0.023], congruency and target emotion word [*F*(2,78) = 0.652, *p* = 0.524, *η*^2^ = 0.016] were not significant. In contrast, we observed a significant cross-over interaction color and target emotion word [*F*(2,78) = 4.687, *p* = 0.012, *η*^2^ = 0.106]. The three-way interaction was not statistically significant [F(2,78) = 0.122, *p* = 0.885, *η*^2^ = 0.003]. However, in the post-hoc pairwise comparison, the response to the sad adjective target was faster in blue than red in the incongruent condition (*t* = 2.625, *p* = 0.012), and the response to happy adjective target was faster in blue compared to red in the incongruent condition (*t* = 2.533, *p* = 0.017). We could not find the significant difference to the angry adjective target (t = 1.300, *p* = 0.198). No significant difference depending on color found in congruent condition.

#### Accuracy

A 2 (color: red or blue) × 2 (congruency: congruent or incongruent, happy, or sad) × 3 (valence of target word: angry, happy, or sad) within-subjects ANOVA revealed no statistically significant main effects of color [*F*(1,39) = 2.669, *p* = 0.110, *η*^2^ = 0.064]. In contrast, the main effects of congruency [F(1,39) = 11.988, *p* = 0.001, *η*^2^ = 0.235] and target word emotion were statistically significant [*F*(2,78) = 12.448, *p* < 0.001, *η*^2^ = 0.242] for accuracy. The interaction effect between color and congruency [F(1,39) = 2.908, *p* = 0.096, *η*^2^ = 0.069], and congruency and target emotion word [F(2,78) = 2.171, *p* = 0.121, *η*^2^ = 0.053] were not significant. In contrast, we observed a significant interaction effect between color and target emotion word [*F*(2,378) = 8.590, *p* < 0.001, *η*^2^ = 0.180]. The three-way interaction was not statistically significant [F(2,78) = 0.062 *p* = 0.940, *η*^2^ = 0.002], but the *post-hoc* pairwise comparison, the response to the sad adjective target was more accurate in blue than red in the incongruent condition (t = 4.000, *p* < 0.001). We could not find the significant difference to the angry adjective target (t = 0.750, *p* = 0.561) nor happy adjective target (*t* = 1.667, *p* = 0.178). No significant difference depending on color found in congruent condition.

We used the facial expressions of the same model (SR) in both experiments. Thus, we also used the repeated-measures ANOVA in both experiments to test whether the accuracy and response time varied across the models. The effect of the model was not significant for either accuracy (*F* = 0.181, *p* = 0.673) or response time (*F* = 0.299, *p* = 0.588).

#### Speed–Accuracy Trade-Off

A correlation between overall speed and accuracy (speed–accuracy trade-off) was not significant (*r* = −0.053, *p* = 0.747).

## Discussion

The current study investigated the effect of color on emotion processing in both semantic and lexical levels. Since pictures and colors have privileged access to the semantic network, and words have privileged access to the lexicon (Glaser and Glaser, 1989), the semantic level should first represent the perception of color stimuli, then the lexical level (Heurley et al., 2013). Therefore, we used a color priming task to examine the facilitation or interruption effect of the color patch preceding the facial expressions to explore the semantic level effect. Then, we used a modified version of the emostroop task (Preston and Stansfield, 2008) to test whether the colors affect the processing of lexical level (emotion word). We observed a significant priming effect of red for angry faces in the Experiment 1. The priming effect indicates semantic level interference of red on angry-related information processing, which is in line with results of previous studies (Moller et al., 2009; Young et al., 2013; Kuniecki et al., 2015; Minami et al., 2018). Red facilitates the response to negative words (Moller et al., 2009), and angry face (Young et al., 2013; Minami et al., 2018). In a similar vein, the event-related potential study using the dot-probe paradigm demonstrated that red-colored negative emotion stimuli captured attention faster and facilitated responses (Kuniecki et al., 2015). However, we could not find the significant priming effect of blue on sad face. Although blue is associated with sadness in many cultures (Byrnes, 1983; Levy, 1984; Clarke and Costall, 2008; Hanada, 2018; Takahashi and Kawabata, 2018), there have been few experimental studies on the effect of the color blue. There is one study showed that cold-related words are detected faster when presented in blue (Lorentz et al., 2016), but not directly on sad.

**Figure 5:**
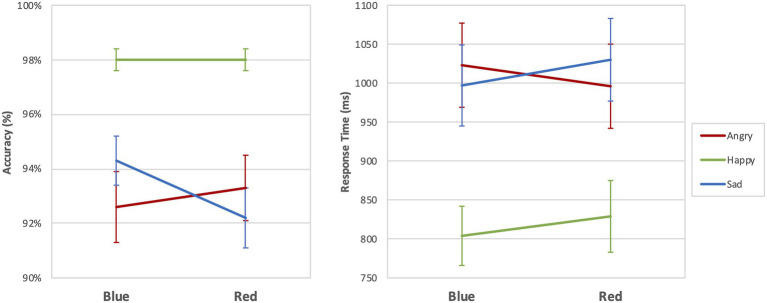
Interaction between color and target word emotion for Experiment 2.

In the color-emostroop task, we observed no significant main effect of color. However, we found significance in the cross-over interaction between color and target word emotion in response time and accuracy (Figure 5). Also, when considering the congruent and incongruent conditions separately, we observed the interference effect of colors only in the incongruent condition. The post-hoc pairwise comparison revealed that respondents detected sad target words faster and more accurately in blue than red in the incongruent condition. On the other hand, they detected the happy target word faster in blue than red. The result suggests the participants may have used color-emotion association as a secondary cue but only in the incongruent condition, where the mismatch between face and word exists.

The results support the associative relationships between colors and specific emotions but with limitations. The study observed semantic interference of red–anger pairs and the cross-over interaction between anger–red and blue–sad pairs at the lexical level. However, the current study also raised several unanswered questions. First, the angry and sad face responses were more accurate when preceded by blue than red in the priming task. The possible distracting effect of red without an emotion processing issue might be a confounding factor. One study showed that red impairs performance in various behavioral tasks and increases avoidance motivation (Elliot et al., 2007). Second, the result of the color-emostroop task shows the effect of blue on the semantic level, but it is unclear that red does not have the effect. The study adopted the initial emostroop paradigm (Preston and Stansfield, 2008) and added color as an additional experimental variable, so it does not contain neutral words as the target. Adding a neutral target might provide additional information about the effect of red on semantic emotion information processing. Third, we found an ambiguous result for blue in the color-emostroop task. Interestingly, the pairwise comparison of response time in the color-emostroop task revealed that a response to a happy target was slower in red than blue in the incongruent condition, similar to the sad target. The result might indicate the possibility of a more complex semantic association of blue. Unlike the common association between red and anger, sometimes, there is an association between blue and positive valence (i.e., Hemphill, 1996; Demir, 2020). Considering a finding on the effect of green on positive word processing (Moller et al., 2009), a further study including green beside red and blue may provide additional answers.

The present study also has some limitations. Although we counterbalanced the order between Experiment 1 and 2 across participants and the effect of model identity was not statistically significant, there may still be an effect of exposure to the model presented for both experiments. Further, the study tested only the effect of red and blue compared to gray as a control condition. Researchers should conduct further studies, including more colors and emotions. Moreover, as perceived colors consist of various physical features (e.g., hue, brightness), future studies are needed to examine the emotional effects associated with these features (Wilms and Oberfeld, 2017).

## Data Availability Statement

The datasets generated for this study are available on request to the corresponding author.

## Ethics Statement

The studies involving human participants were reviewed and approved by Institutional Review Board of Korea University. The patients/participants provided their written informed consent to participate in this study.

## Author Contributions

JK and H-KY conceived and designed the experiments. JK and YP conducted the experiments and analyzed the data. All authors contributed to the article and approved the submitted version.

## Funding

The study was supported by the National Research Fund (NRF-2014R1A1A2057756, NRF-2017M3C7A1041825, and NRF-2020R1A2C1008072).

## Conflict of Interest

The authors declare that the research was conducted in the absence of any commercial or financial relationships that could be construed as a potential conflict of interest.

## Publisher’s Note

All claims expressed in this article are solely those of the authors and do not necessarily represent those of their affiliated organizations, or those of the publisher, the editors and the reviewers. Any product that may be evaluated in this article, or claim that may be made by its manufacturer, is not guaranteed or endorsed by the publisher.
